# Diagnosis and management of food-induced anaphylaxis

**DOI:** 10.1186/2045-7022-1-S1-S64

**Published:** 2011-08-12

**Authors:** Dana Wallace

**Affiliations:** 1 Nova Southeastern University, Fort Lauderdale, Florida, USA

## 

Anaphylaxis is an acute, life-threatening, systemic reaction with varied clinical presentations and severity that results from the systemic release of mediators from mast cells and basophils. The diagnosis of food-induced anaphylaxis is based on a combination of medical history and presence of specific IgE to the responsible food. In general, the more rapid the onset of symptoms following exposure to the allergen, the more likely the reaction will be severe and life threatening. However when ingested food is the allergen, it may take up to 25 minutes from onset of anaphylaxis to cardiopulmonary arrest, making the diagnosis even more challenging. In food-induced, fatal anaphylaxis episodes up to 80% are not associated with cutaneous signs or symptoms, the signal that most patients will recognize first. Furthermore, it is well established that patients with food-induced anaphylaxis under utilize epinephrine. Thus, ongoing education for the patient who is at risk for anaphylaxis becomes essential.

While occurrences of food allergy are believed to be increasing, the true prevalence is unknown. Food allergies are highest in children and adolescents and are thought to be the most common cause of anaphylaxis in the outpatient setting, accounting for approximately 30% of fatal reactions. Fatalities from food-induced anaphylaxis are highest in teenagers and young adults, among those with asthma, and in cases when there has been a delay in the administration of epinephrine. Biphasic reactions occur in approximately 25% of fatal and near-fatal, food-induced anaphylaxis events. Special circumstances involving food allergy that will be discussed include exercise-induced anaphylaxis, unsuspecting hidden food allergy, and the significance of food allergy for vaccine administration.

The treatment of anaphylaxis involves the immediately administration of epinephrine. When there is any doubt about the diagnosis, it is generally best to administer epinephrine rather than waiting for more severe symptoms. In the treatment of food-induced anaphylaxis, more than a single dose of epinephrine may be required; these doses can be given every 5-10 minutes, and a longer period of observation is recommended. While IM administration is preferred by many national and international guidelines, the controversy over IM vs. subcutaneous epinephrine use will be discussed. Following the administration of epinephrine, other treatment modalities include 1) placing the patient in a supine position, 2) oxygen, and 3) fluid replacement. The role of the second line, supportive medications including H1 and H2 antihistamines and corticosteroids has not been established for the treatment of acute, biphasic, or prolonged anaphylaxis. Glucagon may be considered in patients taking a beta-blocker who are not responding to epinephrine.

Physicians and office staff need to maintain clinical proficiency in anaphylaxis management, which should include an established written plan to deal with anaphylaxis and regular practice sessions. An action plan for the diagnosis and management of food-induced anaphylaxis should be provided to every patient with food allergies. A review of the action plan and technique for auto-injection of epinephrine should occur at regularly scheduled office visits. Recognizing that at the present time, there is no way to prevent food-induced anaphylaxis other than by avoiding the food. the patient requires frequent reinforcement that epinephrine, NOT antihistamines, is the only effective medication for the treatment of anaphylaxis and that a delay in administration is associated with more severe symptoms and a higher rate of fatality. Every patient with food-induced anaphylaxis must always have at least two doses of auto-injectable epinephrine immediately available.

